# Genetic and metabolic inflammation signatures in chronic inflammatory demyelinating polyneuropathy: the role of *IL18* polymorphisms and short-chain fatty acids

**DOI:** 10.3389/fnmol.2025.1738817

**Published:** 2026-01-12

**Authors:** Szymon Andrusiów, Marta Dratwa-Kuzmin, Piotr Łacina, Patrycja Bochen, Klaudia Gładysz, Bogumiła Szponar, Magdalena Koszewicz, Katarzyna Bogunia-Kubik

**Affiliations:** 1 Department of Neurology, Faculty of Medicine, University Centre of Neurology and Neurosurgery, Wroclaw Medical University, Wroclaw, Poland; 2 Laboratory of Clinical Immunogenetics and Pharmacogenetics, Hirszfeld Institute of Immunology and Experimental Therapy, Polish Academy of Sciences, Wroclaw, Poland; 3 Laboratory of Medical Microbiology, Hirszfeld Institute of Immunology and Experimental Therapy, Polish Academy of Sciences, Wroclaw, Poland; 4 Department of Chemistry, University of Wroclaw, Wroclaw, Poland; 5 Clinical Neurophysiology Laboratory, Faculty of Medicine, University Centre of Neurology and Neurosurgery, Wroclaw Medical University, Wroclaw, Poland

**Keywords:** chronic inflammatory demyelinating polyneuropathy, IL18 promoter polymorphisms, inflammatory cytokines, nerve conduction studies, short-chain fatty acids

## Abstract

**Introduction:**

Chronic inflammatory demyelinating polyneuropathy (CIDP) remains diagnostically challenging, with limited biological markers to aid phenotyping and differential diagnosis, particularly at the CIDP–diabetes mellitus (DM) interface.

**Methods:**

We investigated inflammatory genetic and metabolic readouts in CIDP by integrating interleukin 18 (*IL-18*) promoter variation with cytokines and short-chain fatty acids (SCFAs). 32 untreated CIDP patients and 15 controls underwent clinical scoring, nerve-conduction studies (NCS), *IL-18* genotyping (rs187238, rs1946518, rs1946519), serum cytokine profiling (IL-2, tumor necrosis factor α (TNF-α), *IL-18*), and SCFA quantification in stool, serum, and cerebrospinal fluid (CSF).

**Results:**

No group-level differences emerged for IL-2, TNF-α, or IL-18 in serum or CSF, and CIDP subgroups (DM+ vs DM−; classical vs atypical) did not differ in NCS severity or electromyography (EMG) denervation. In contrast, *IL18* promoter variation showed various associations: rs1946518 G allele correlated with peroneal nerve shorter compound motor action potential (CMAP) distal latency and lower ulnar nerve sensory nerve action potential (SNAP) amplitude. Additionally, carriers of the rs187238 C allele showed significantly higher CSF protein concentrations, whereas the rs1946518 G allele was associated with a trend toward lower CSF protein levels. Moreover, the rs187238 C and rs1946518 T alleles were associated with lower CSF butyrate levels. A haplotype analysis indicated that GGG (rs187238, rs1946518, rs1946519) aligned with shorter peroneal nerve CMAP distal latency, lower disability (INCAT), and a lower CSF protein, whereas *CTT* associated with higher CSF protein and lower CSF butyrate concentrations. We confirmed the presence of acetate, propionate, and butyrate in human CSF and demonstrated serum–CSF equivalence for these SCFAs, while stool concentrations were higher, as expected.

**Discussion:**

Collectively, *IL18* polymorphisms and SCFAs readouts emerge as biologically grounded candidates for patient stratification in CIDP; these findings warrant validation in larger, multicenter cohorts integrating electrophysiology with CSF/serum biomarkers and microbiome profiling.

## 1 Introduction

Chronic inflammatory demyelinating polyneuropathy (CIDP) is a rare disease whose epidemiology varies geographically, with an incidence ranging from 1.61/100,000 in the Japanese population (Iijima et al., 2008), through 7.7/100,000 in Norway (Mygland and Monstad, 2001) to 8.9/100,000 in the United States (Laughlin et al., 2009). In the typical form, it manifests as progressive, symmetrical paresis of both proximal and distal muscles and sensory disturbances, mainly in the form of paresthesias and hypersensitivity in a sock-and-glove distribution (Van den Bergh et al., 2021). In addition to the classical form, there is a group of atypical variants, differing in the distribution of the disorders, limited to one type of nerve fiber or presenting with a different onset (acute/chronic) (Menon et al., 2021; Doneddu et al., 2019). Furthermore, a wide range of autoimmune, infectious, metabolic, and toxic conditions are capable of inducing peripheral nerve demyelination and warrant careful consideration in the differential diagnosis of CIDP (Cox and Gwathmey, 2021; Ercolini and Miller, 2009), as reflected in recent guidelines for the diagnosis and treatment of CIDP (Van den Bergh et al., 2021). Diabetes mellitus (DM) represents one of the principal conditions in this context, as it is highly prevalent and can contribute to peripheral nerve demyelination (Andrusiów et al., 2023). Epidemiological studies carried out to date, have given conflicting results on the prevalence of CIDP and DM co-occurrence. However, more recent studies seem to support a higher prevalence of DM in patients with CIDP, especially in the elderly (Laughlin et al., 2009; Bril et al., 2016; Rajabally et al., 2017; Rajabally et al., 2020). The difficulty in the differential diagnosis of CIDP from DM is well known, due to the coincidence of clinical manifestations and electrophysiological findings (Latov, 2011; Dunnigan et al., 2013a; Abraham et al., 2015). It appears that the single diagnostic label of CIDP currently encompasses several distinct patient populations, as well as a subset of individuals who are misdiagnosed with other primary or secondary neuropathies, most notably diabetic polyneuropathy (Allen and Lewis, 2015; Moritz et al., 2021). The present considerations are important because demyelinating polyneuropathy in diabetes does not respond well to immunomodulatory treatment (Breiner et al., 2019). Furthermore, the co-occurrence of diabetes in patients with CIDP also appears to worsen the chances of treatment efficacy (Andrusiów et al., 2023). The search for a good method of differentiating neuropathy in the course of the above-mentioned disease entities is ongoing (Lotan et al., 2015; Dunnigan et al., 2013b; Heiling et al., 2024; Jann et al., 2003). There is currently no recognized biomarker that is unequivocally useful in the differential diagnosis, as reflected in the criteria for the diagnosis of CIDP, which are mainly based on clinical and electrophysiological diagnosis (Van den Bergh et al., 2021). Diagnostic and prognostic biomarkers can take many forms. They may be newly discovered biochemical molecules or measurement methods, or they may be a new interpretation of those already discovered (Allen et al., 2021; Brannagan, 2011). In CIDP, imaging readouts such as quantitative magnetic resonance neurography and nerve ultrasound have shown associations with clinical and electrophysiological burden and can aid differential diagnosis (Heiling et al., 2024; Preisner et al., 2023). Also methods that extend conventional nerve-conduction studies (NCS) and electromyography (EMG) are being refined to capture features invisible to standard studies (Tiric-Campara et al., 2014; Hazeena et al., 2022). Among biochemical biomarkers, cerebrospinal-fluid sphingomyelin has been proposed as a diagnostic and staging marker of acquired demyelinating neuropathies, including CIDP (Capodivento et al., 2021). Genetic stratifiers are also under active investigation: cytokine-gene polymorphisms (e.g., interleukin 10 (IL-10), IL-6) have been linked to CIDP susceptibility/severity in exploratory cohorts (Bozovic et al., 2023).

In the majority of patients, pathological antibodies or a secondary cause of neuropathy cannot be found and they are classified as idiopathic (Shastri et al., 2023; Pascual-Goñi et al., 2024). The disease is caused by demyelination of the peripheral nerves with an unclear pathogenesis and an unknown causative factor (Dyck and Tracy, 2018). Experimental studies suggest a loss of central tolerance by CD4 + T cells, which initiates a cascade of autoimmune reactions (Su et al., 2012). A role for macrophages and CD8+lymphocytes has been postulated as factors involved in direct damage to the blood-nerve barrier and myelin sheath (Koike et al., 2018; Vallat et al., 2020; Schneider-Hohendorf et al., 2012; Yang et al., 2015). The role of the costimulatory molecule B7.2 (CD86) in the induction of neuritis has been demonstrated in experimental models (Salomon et al., 2001; Bour-Jordan et al., 2013). Numerous other factors have also been taken into account, such as the role of Treg lymphocytes, the complement system or yet-unidentified pathological antibodies (Shastri et al., 2023; Wolbert et al., 2020). In contrast, there is still no direct causative factor that triggers an autoimmune reaction. Autoantigens of peripheral nerves that are targets for pathological antibodies, such as anchoring junction components, are currently being sought (Moritz et al., 2021). Future discoveries of pathological antibodies will probably lead to the separation of specific disease entities from the idiopathic CIDP group, as has been done with nodopathies and anti-myelin-associated glycoprotein (MAG) neuropathy (Pascual-Goñi et al., 2024; Querol et al., 2017; Ogata, 2020; Broers et al., 2023), which are included as a separate disease according to the latest guidelines (Van den Bergh et al., 2021). In this context, it seems essential to search for factors that could serve as biological markers useful for diagnosis or for prognostication in patients with CIDP. One promising avenue is the evaluation of the utility of inflammatory cytokines in this regard. Particularly relevant may be the role of IL-2 and tumor necrosis factor *α* (TNF-α), given their pleiotropic effects on inflammatory responses and their description as potential pathogenic contributors in CIDP (Misawa et al., 2001; Dziadkowiak et al., 2021).

In recent years, interest in the impact of gut microbiota on neurological diseases has grown exponentially (Cryan et al., 2020). One of the areas being studied is microbiota metabolism. Short-chain fatty acids (SCFAs) are a group of gut bacterial metabolites that are thought to affect the human body through a variety of mechanisms. They are an important nutrient for enterocytes, stabilize the intestinal barrier by regulating the composition of the intestinal microbiota and have immunomodulatory effects on intestinal wall leukocytes (Blacher et al., 2017). Emerging evidence indicates that SCFAs, described as key metabolites produced by gut microbiota, can modulate the integrity of both the blood–brain barrier and the blood–cerebrospinal fluid (CSF) barrier (Xie et al., 2023; Chenghan et al., 2025). SCFAs have been shown to reinforce tight junctions at these barrier sites, thereby reducing permeability and potentially limiting the infiltration of pro-inflammatory cells or molecules into the central nervous system (CNS) (Xie et al., 2023). In parallel, SCFAs exert broad immunomodulatory effects on the host’s systemic immune responses. For example, butyrate and propionate can regulate the production of cytokines by neutrophils, macrophages, dendritic cells, and lymphocytes, skewing the immune system toward an anti-inflammatory profile (Du et al., 2024). Reduced levels of SCFAs have been described in multiple sclerosis, as an inflammatory CNS disease (Montgomery et al., 2024). Studies demonstrating the involvement of SCFAs in inflammatory neuropathies are limited and so is information on the possibility of SCFA penetration into the CSF (Deng et al., 2019). In conclusion, despite the numerous reports on the effects of SCFAs in human physiology and pathophysiology, high-quality evidence in human studies is still lacking (Dalile et al., 2019). Consequently, recent studies have started to measure fecal SCFA levels as accessible indicators of gut microbiota activity that might influence disease processes in neuroinflammatory disorders. This approach is being applied as an alternative to broad microbiome analyses, for instance to investigate whether altered SCFA profiles are associated with neuroinflammatory conditions (Schwerdtfeger et al., 2025; Becker et al., 2021). One cytokine that may significantly modulate the effect of gut microbiota derived metabolites is IL-18. This molecule, produced downstream of the NLRP6 inflammasome, appears to have important effects on the intestinal barrier, inflammatory processes within the intestinal wall and regulation of the intestinal microbiome (Seregin et al., 2016; Yasuda et al., 2019). This molecule also has broad pleiotropic effects (Yasuda et al., 2019; Ihim et al., 2022), being a pro-inflammatory molecule and influencing neuronal survival by increasing the expression of brain-derived neurotrophic factor (BDNF) (Zhou et al., 2014). Despite numerous studies, the role that IL-18 plays in autoimmune processes remains unclear, and research findings often remain contradictory (Nowarski et al., 2015; Salcedo et al., 2010; Elinav et al., 2011). Numerous studies indicate increased IL-18 concentrations in a number of autoimmune processes, including the CNS (Ihim et al., 2022). Isolated reports also indicate a role for IL-18 in the pathogenesis of inflammatory demyelinating neuropathies (Jander and Stoll, 2001). In this context, particular importance may be attributed to functional promoter polymorphisms of *IL18*: −137*G*/*C* (rs187238) and −607*C*/*A* (rs1946518; in LD with rs1946519). Expression analyses indicate that these variants modulate *IL18* transcription, altering the secretory potential of cells and the organism’s inflammatory set point. These effects are context-dependent and influenced by population background, yet associations between promoter genotypes/haplotypes, IL-18 levels, and phenotypic traits in autoimmune diseases have been repeatedly documented. The rs187238 and rs1946518/rs1946519 SNPs are common functional promoter variants that modulate *IL18* transcription and IL-18 release and have been repeatedly associated with altered susceptibility or clinical expression of autoimmune and chronic inflammatory diseases (Dziedziejko et al., 2012; Ramezanzadeh et al., 2024; Chen et al., 2012). Accordingly, rs187238/rs1946518 may be viewed as determinants of disease severity and clinical presentation (Dziedziejko et al., 2012; Ramezanzadeh et al., 2024; An et al., 2008). In this context, *IL18* promoter genotypes and haplotypes can be viewed as generic immune-response modifiers rather than disease-specific mutations, providing a biologically grounded rationale to investigate their influence on demyelinating and barrier-related phenotypes even in a rare neuroimmune disorder such as CIDP (Ihim et al., 2022; Dziedziejko et al., 2012; Ramezanzadeh et al., 2024).

## 2 Aim

This study aimed to investigate the gut microbiota–immune system–nerve axis in CIDP by integrating three complementary layers: genetic variation in the *IL18* promoter, systemic inflammatory cytokines (IL-2, TNF-*α*, IL-18), and SCFAs quantified in stool, serum and CSF. Specifically, we sought to assess associations between *IL18* genotypes/haplotypes, cytokine and SCFA readouts and clinical/electrophysiological parameters in CIDP. Furthermore, we also aimed to explore whether these biomarkers differ across clinically relevant CIDP subgroups, including patients with and without coexisting DM. Collectively, this integrated approach was intended to yield biologically grounded markers that could support patient stratification and improve differential diagnostic assessment at the CIDP–diabetes interface.

## 3 Materials and methods

### 3.1 Populations

Data were collected from 32 patients who were hospitalized in the Neurological Department, Wroclaw Medical University between 2021 and 2024. The study was approved by the Bioethical Committee at Wroclaw Medical University (ethical approval code: 932/2021) and has been performed in accordance with the ethical standards laid down in the 1964 Declaration of Helsinki and its later amendments. Patients included in the study had been diagnosed with CIDP during hospitalization and had not been previously treated with immunomodulatory therapy. The broad differential diagnosis was made in accordance with the European Federation of Neurological Societies (EFNS) and the Peripheral Nerve Society (PNS) guidelines for CIDP from 2021 (Van den Bergh et al., 2021). Evidence of central nervous system involvement on brain MRI performed as part of the differential diagnostic work-up constituted an exclusion criterion. All patients were routinely assessed using Inflammatory Neuropathy Cause and Treatment (INCAT) disability score.

In the CIDP group, 14 (44%) patients had diabetes mellitus (DM+), and nine (28%) had other specified comorbidities, including chronic hepatitis B virus infection, heart failure, chronic kidney disease, rheumatoid arthritis, hypothyroidism, hyperthyroidism, interstitial lung disease, ischaemic stroke and depression. Among patients with diabetes, one had type 1 DM and 13 had type 2 DM. Eight (25%) patients had atypical CIDP (multifocal, motor-predominant or sensory-predominant). In the study group, the mean age was 63 years (range 40–84 years). The duration of the disease was between 6 months up to 26 years. The control group consisted of 15 patients hospitalized at the clinic for other reasons (including headaches and normotensive hydrocephalus) without clinical and/or electrophysiological features of neuropathy. In the control group the mean age was 57 years (range 21–79 years). A detailed description of both the CIDP and control groups are included in Table 1.

**Table 1:** Demographic and clinical characteristics of the CIDP group and control group.

Characteristics	CIDP group (*n* = 32)	Control group (*n* = 15)
Mean age (years)	61 (40–84)	57 (21–79)
Sex (male/female)	27/5	7/8
Disease duration (years)	0.5–26	-
Comorbidities (*n*)	9	9
Comorbidities (type)	Ischaemic stroke/Hyperthyroidism/Depression/ HBV/Heart failure/ Chronic kidney disease/Rheumatoid arthritis/Hypothyroidism/Interstitial lung disease	Headaches/Normotensive hydrocephalus
Diabetes mellitus (*n*)	14	1
Atypical CIDP (*n*)	8	-

**Figure 1:**
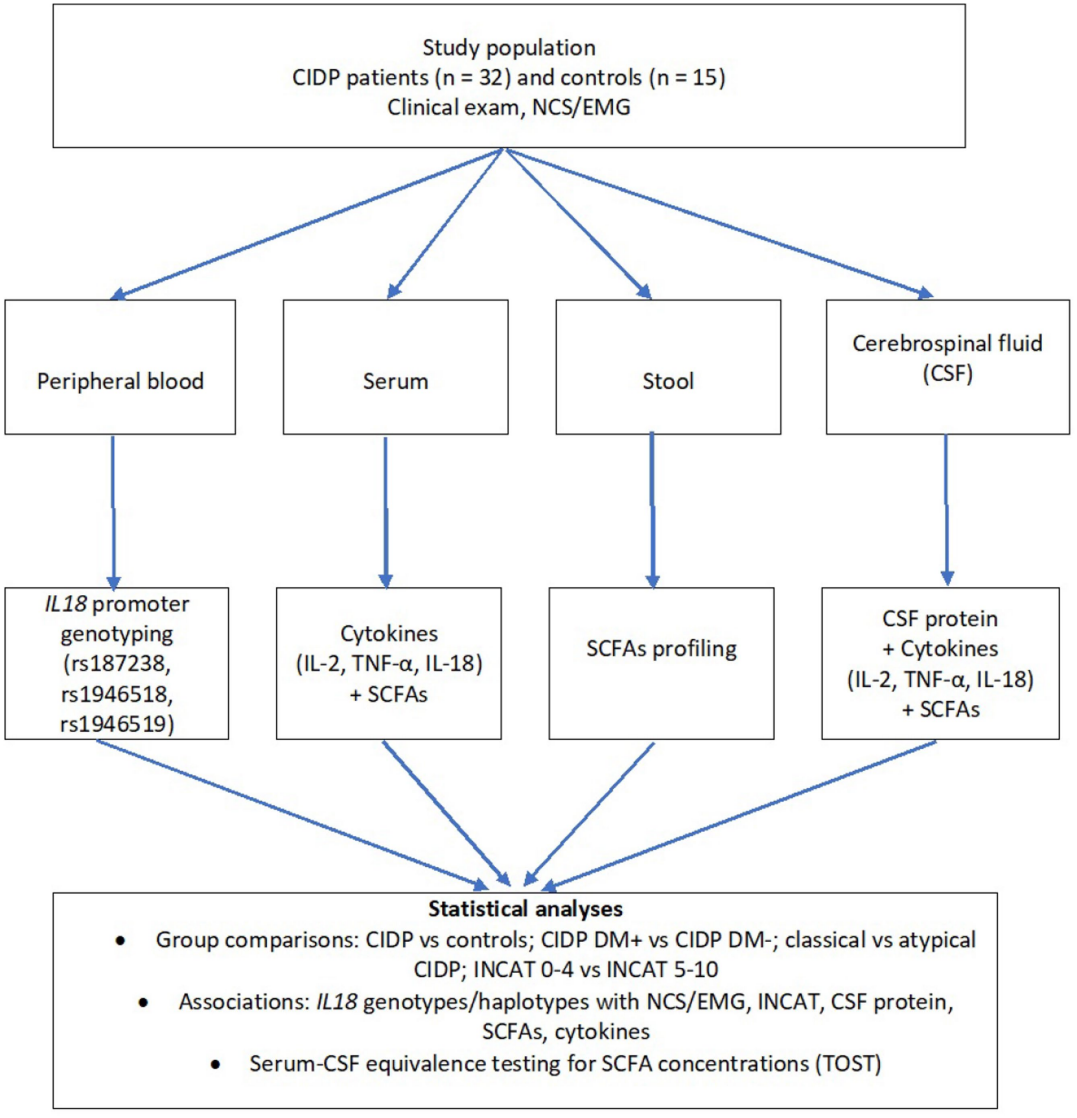
Study design and analytical workflow. Overview of patient recruitment, biospecimen collection, and downstream analyses, together with subsequent statistical analyses in CIDP patients and controls.

The successive stages of the study, along with the types of biological material collected and its downstream use, are presented in Figure 1.

### 3.2 Electrophysiological studies

Standard electrophysiological assessments included: distal latency (DL), compound motor action potential (CMAP) amplitude, motor nerve conduction velocity (MCV), F-wave latency (F-Lat), sensory nerve action potential (SNAP) amplitude, and sensory nerve conduction velocity (SCV). Conduction studies were performed in the median, ulnar, peroneal, tibial and sural nerves. Sensory nerve conduction tests were carried out using the antidromic technique. Electromyography of the tibialis anterior muscle was also conducted. All studies were performed using the Viking Quest system, version 10.0. For each patient, the same nerve was examined under standardized conditions, including a fixed distance between the stimulating cathode and the active recording electrode, with stimulation applied at a standardized site to evaluate distal onset latency, amplitude, and conduction velocity. The electrical stimulus duration was 0.2 ms for motor fibers and 0.1 ms for sensory fibers. The ambient room temperature was maintained between 21 and 23 °C, with hand temperatures not falling below 32 °C and leg temperatures not below 30 °C. Examinations were conducted according to the method described by Oh (2003) experienced, certified clinicians, ensuring that all procedures complied with the 2021 EFNS/PNS guidelines for CIDP (Van den Bergh et al., 2021).

### 3.3 Cerebrospinal fluid collection

Cerebrospinal fluid was collected via lumbar puncture at the L3/L4 intervertebral space under sterile conditions, following standard clinical procedures. CSF samples were then analyzed for routine parameters, including cell count, protein concentration, and glucose levels, as well as for immunological and biomarker assessments relevant to CIDP.

### 3.4 DNA analysis

DNA was isolated from peripheral blood samples collected in EDTA tubes using the NucleoSpin Blood DNA Purification Kit (Macherey-Nagel, Germany), following the manufacturer’s instructions. *IL18* genotyping was performed using the LightSNiP assay (TIB Molbiol, Germany). Amplifications were performed on a LightCycler480 II Real-Time PCR system (Roche Diagnostics International AG, Rotkreuz, Switzerland) according to the recommendations of the manufacturer. The PCR protocol consisted of an initial denaturation at 95 °C for 10 min, followed by 45 cycles of 95 °C for 10 s, 60 °C for 10 s, and 72 °C for 15 s. This was followed by a cycle of 95 °C for 30 s and 40 °C for 2 min, after which a gradual melting phase from 75 °C to 40 °C was performed. Genotypes were determined by analyzing the melting temperatures and melting curve profiles.

### 3.5 Cytokine measurement

The assays were performed in blood serum and CSF. Following this, the blood was subjected to a centrifugal process at 30 min of incubation time at ambient temperature. Serum concentrations of IL-2, IL-18, and TNF-*α* (expressed in pg/mL) were quantified using the Human ProcartaPlex Mix & Match Panels (Thermo Fisher Scientific, USA) on the Luminex 200 system (Luminex Corp., USA). All procedures were strictly adhered to, in accordance with the manufacturer’s protocol. The median fluorescence intensity (MFI) measured from the beads was then compared against a standard curve using xPonent 4.2 software to compute the analyte concentrations in pg/mL. The concentrations present within the samples were determined by applying a 5-parameter logistic fit to the MFI, subsequently converting them into precise analyte levels.

### 3.6 Short-chain fatty acids measurement

A 500 μL aliquot of stool sample was mixed with 3 mL of 70% ethanol for extraction. After centrifugation to remove any solid debris, 500 μL of the resulting supernatant was transferred into a new tube. To this, several reagents were added: 50 μL of an internal standard solution (2-ethylbutyric acid at 200 mM in 50% aqueous methanol), 300 μL of dehydrated pyridine (Merck) at a 3% v/v concentration in ethanol, 300 μL of a 250 mM solution of N-(3-dimethylaminopropyl)-N′-ethylcarbodiimide hydrochloride (Sigma-Aldrich) in ethanol, and 300 μL of a 20 mM solution of 2-nitrophenylhydrazine hydrochloride (Sigma-Aldrich) in ethanol. The mixture was then incubated at 60 °C for 20 min. To stop the reaction, 200 μL of a sodium hydroxide solution (15% w/v in water, prepared at an 80:20 v/v ratio of sodium hydroxide solution to methanol) was added. After allowing the sample to cool, it was extracted twice by adding 2 mL of a 0.5 M phosphoric acid aqueous solution together with 4 mL of diethyl ether each time. The organic phase obtained from these extractions was collected and subsequently washed with water. This organic layer, which contained the fatty acid hydrazides, was then evaporated to dryness, reconstituted in 150 μL of methanol, and prepared for high-performance liquid chromatography (HPLC) analysis.

HPLC was carried out using a 1,525 Binary HPLC Pump coupled with a 2,489 UV/Visible Detector (Waters). Separation was achieved on the C18 column (Shimadzu 5 μm C18-120 Å, 250 × 4.6 mm) using a mobile phase composed of acetonitrile, methanol, and water in the ratio of 30:16:54. The column was maintained at 50 °C, with a flow rate of 1 mL/min, and detection was performed at a wavelength of 400 nm.

For serum and CSF samples, a similar protocol was followed with minor modifications. In these cases, the samples were extracted using 1 mL of 70% ethanol, and CSF samples were processed without a centrifugation step.

### 3.7 Data analysis

*IL18* SNP genotyping results were tested for deviation from the Hardy–Weinberg equilibrium using Haploview software, v. 4.2. For each experiment, data normality was verified with the Shapiro–Wilk test. Considering that distribution of some data deviated from normal distribution, the non-parametric *U* Mann–Whitney test was performed; for normally distributed data, the Student’s *t*-test was applied. For data visualization the Tukey method was used. All of these analyses were performed with the Real Statistics Resource Pack for Microsoft Excel 2013 (version 15.0.5023.1000, Microsoft, Redmont, Washington, USA) and GraphPad Prism (version 8.0.1, GraphPad Software, San Diego, CA, USA). The paired TOST equivalence testing between fatty acid concentrations in serum and CSF were performed using RStudio (Posit, PBC, Boston, MA, USA) and the TOSTER package. *p*-values lower than 0.05 were considered statistically significant, while those between 0.05 and 0.10 as indicative of a trend.

## 4 Results

In an extensive analysis, comparing the study and control groups and, within the CIDP cohort, between DM+ and DM− patients, and between classical and atypical CIDP, no statistically significant differences were found in the concentrations of the assessed cytokines (TNF-*α*, IL-2, IL-18) in either serum or CSF. The patient subgroups also did not differ significantly with respect to the severity of abnormalities on NCS (median, ulnar, peroneal, tibial, sural nerves) or the presence of active or chronic denervation on EMG. The following analyses were performed in the entire cohort, without stratification into the previously described subgroups.

**Figure 2:**
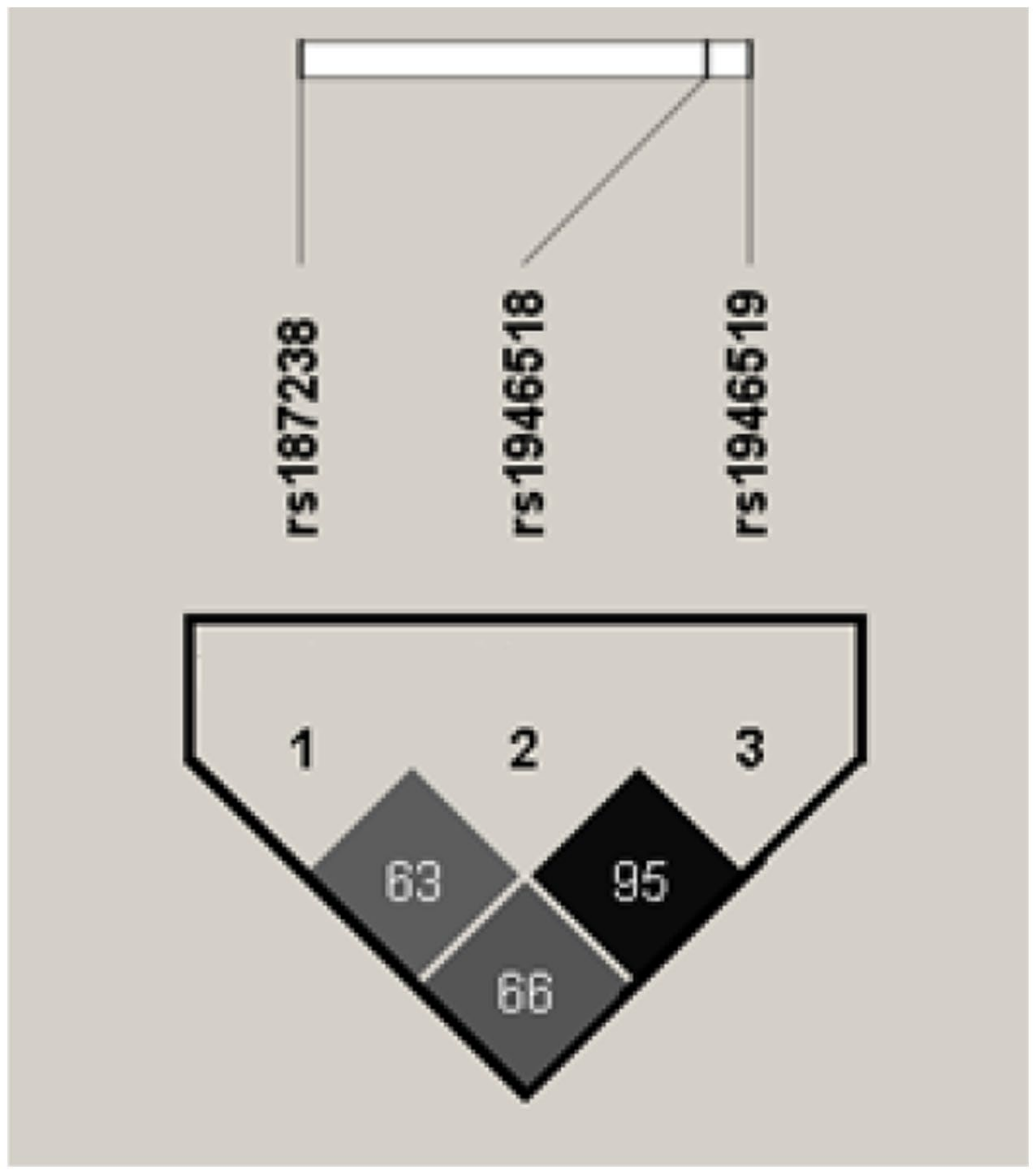
Linkage disequilibrium between the three *IL18* SNPs under study. Darker color represents higher r^2^ values, and the value shown inside each square is r^2^x10^2^. Image generated by Haploview (Barrett et al., 2005).

**Figure 3:**
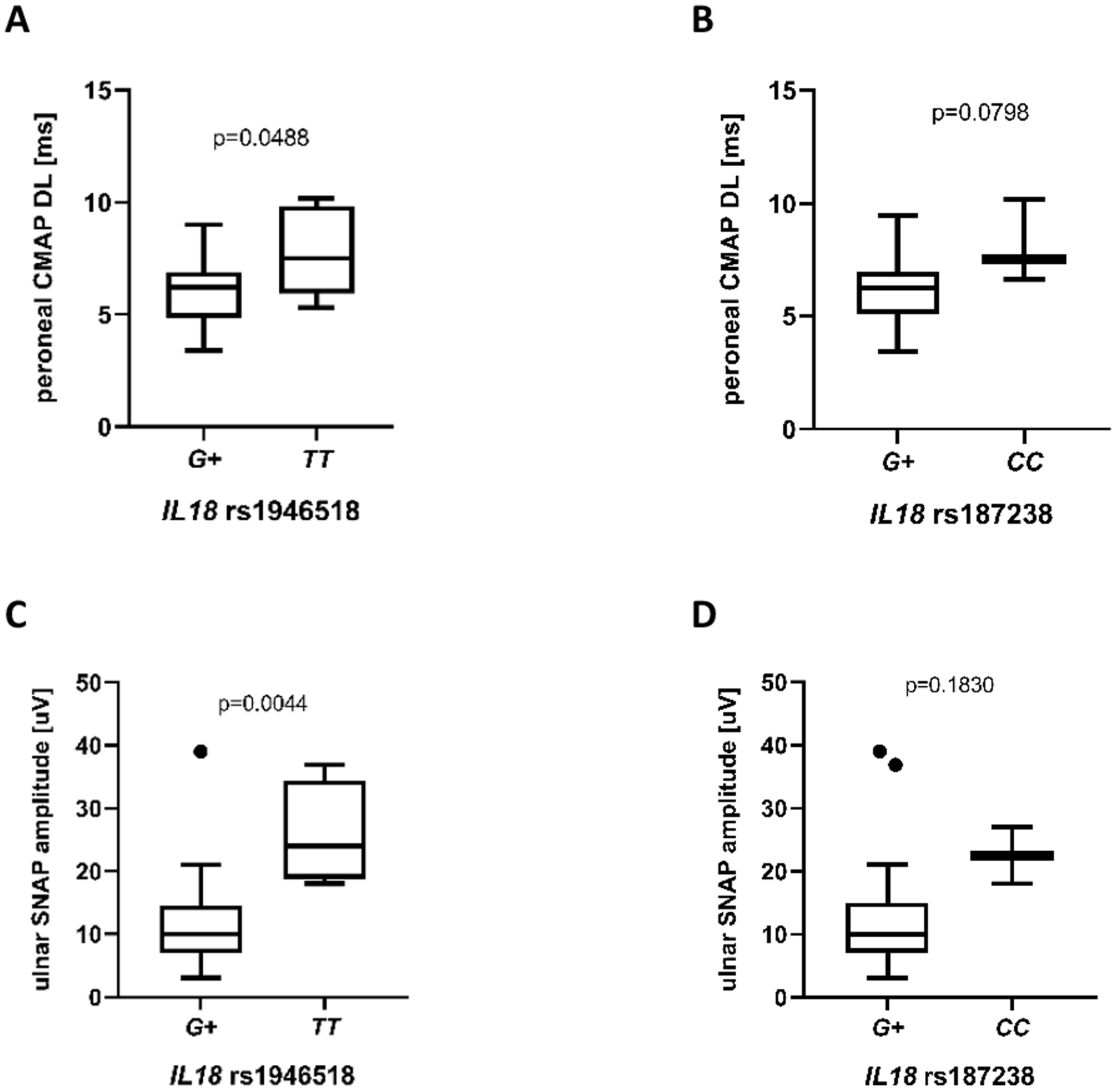
Peroneal CMAP distal latency (ms) **(A,B)** and ulnar SNAP amplitude (μV) **(C,D)** in patients with different *IL18* genotypes in rs1946518 **(A,C)** and rs187238 **(B,D)**. In all panels, “*G+*” indicate carriers of the respective allele in either the homozygous or heterozygous state, whereas “*TT*” and “*CC*” denote homozygosity for the *T* and *C* allele, respectively. Group sizes and statistical tests were as follows: **(A)** *G +* vs. *TT*, *n* = 20 vs. *n* = 5, unpaired *t*-test; **(B)** *G +* vs. *CC*, *n* = 22 vs. *n* = 3, unpaired *t*-test; **(C)** *G +* vs. *TT*, *n* = 21 vs. *n* = 4, Mann–Whitney U test; **(D)** *G +* vs. *CC*, *n* = 23 vs. *n* = 2, Mann–Whitney U test. Data are presented as box plots and expressed as **(A,B)** mean ± standard deviation and as **(C,D)** median ± interquartile range; individual dots represent single observations.

In the next step, we analyzed the impact of the selected *IL18* SNPs on clinical, electrophysiological, and laboratory parameters. Genotyping results were not available for two patients due to technical limitations. The distribution of *IL18* genotypes for the analyzed polymorphisms (rs187238, rs1946518, rs1946519) is presented in Table 2. As rs1946518 and rs1946519 were found to be in almost complete linkage disequilibrium (r^2^ = 0.95, Figure 2), only one of them (rs1946518) was selected to be included in further analyses. We observed associations with motor conduction, expressed as changes in conduction within the peroneal nerve, as well as with sensory conduction, expressed as changes in ulnar nerve conduction. The *IL18* rs1946518 *G* allele (genotypes *GG* and *TG*) was associated with a significantly shorter distal CMAP latency in the peroneal nerve, as compared to *TT* genotype (mean: 6.160 vs. 7.816 ms, *p* = 0.0488; Figure 3A). A similar relationship was observed for the rs187238 *G* allele (genotypes *GG* and *GC*), although this was only a trend, as compared to *CC* genotype (mean: 6.272 vs. 8.100 ms, *p* = 0.0798; Figure 3B). The rs1946518 *G* allele was associated with a lower ulnar SNAP amplitude, as compared to *TT* genotype (median 10.00 vs. 24.00 μV; *p* = 0.0044 Figure 3C). No such associations were detected for the rs187238 *G* allele, as compared to *CC* genotype (median: 10.00 vs. 22.50 μV; *p* = 0.1830 Figure 3D).

**Table 2:** Genotypes and haplotypes frequencies of *IL18* promoter polymorphisms (rs187238, rs1946518, rs1946519) in CIDP patients and controls.

*IL18* genetic polymorphism	CIDP group frequency (*n* = 30)	Control group frequency (*n* = 15)
rs187238		
*GG*	15 (50%)	8 (53.3%)
*CG*	12 (40%)	7 (46.7%)
*CC*	3 (10%)	0
rs1946518		
*GG*	12 (40%)	7 (46.7%)
*GT*	12 (40%)	6 (40%)
*TT*	6 (20%)	2 (13.3%)
rs1946519		
*GG*	13 (43.3%)	7 (46.7%)
*TG*	11 (36.7%)	6 (40%)
*TT*	6 (20%)	2 (13.3%)
Haplotype *GGG*	24 (80%)	13 (86.7%)
Haplotype *CTT*	15 (50%)	7 (46.7%)

As we found all the three *IL18* SNPs to be in relatively high linkage disequilibrium (Figure 1), we decided to investigate the most common haplotypes, *GGG* and *CTT* (the order of alleles is: (1) rs187238, (2) rs1946518, (3) rs1946519). The *GGG* haplotype was associated with shorter distal CMAP latency in the peroneal nerve (mean: 6.16 vs. 7.816 ms, *p* = 0.0488), lower ulnar SNAP amplitude (median 10 vs. 24 μV, *p* = 0.0094), and milder clinical manifestations as assessed by the INCAT scale (*p* = 0.0413).

**Figure 4:**
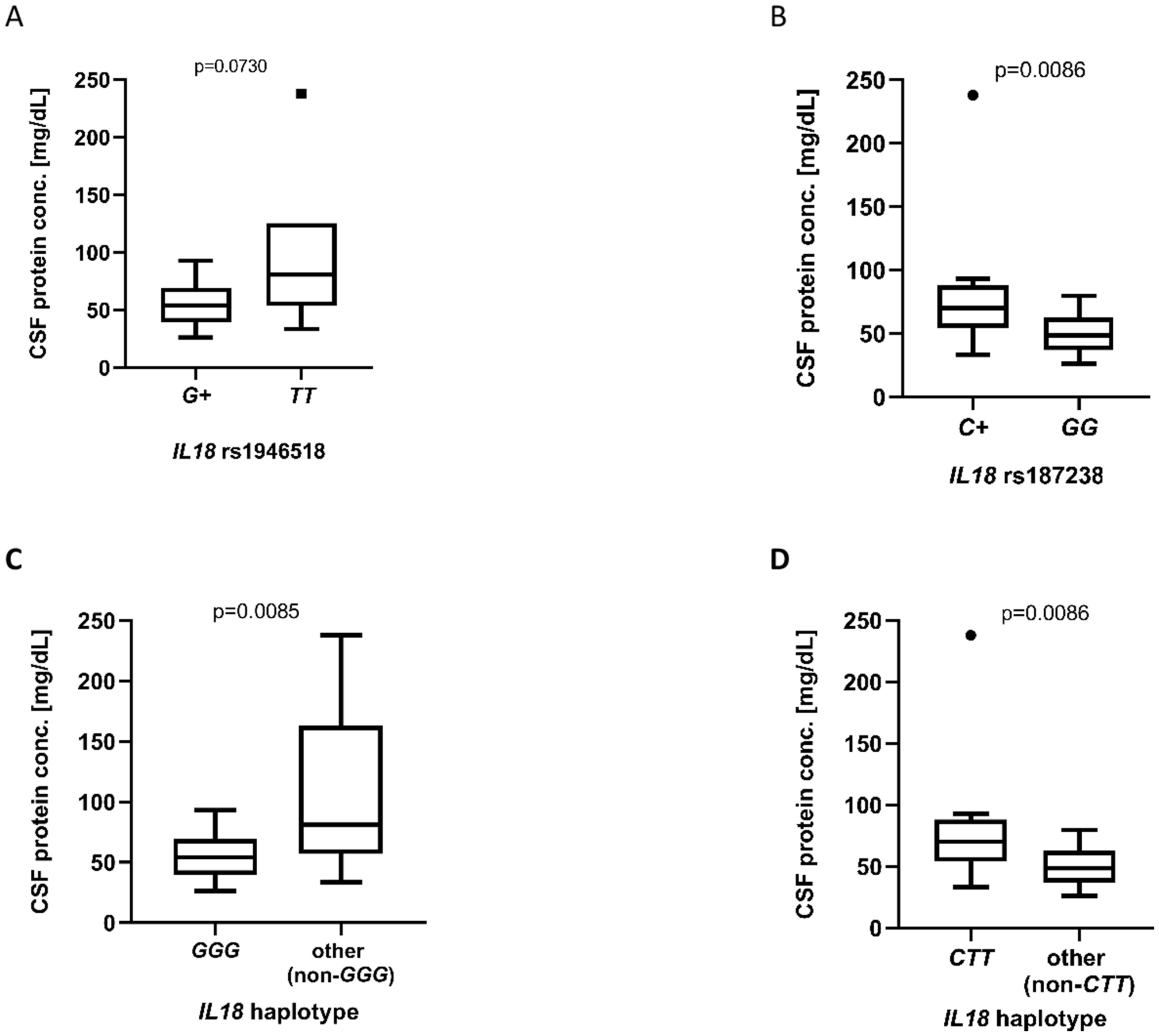
Cerebrospinal fluid protein concentrations (mg/dL) in patients with different *IL18* polymorphic variants: rs1946518 **(A)**, rs187238 **(B)**, and with versus without the *GGG* **(C)** or *CTT* **(D)** haplotype (allele order: rs187238, rs1946518, rs1946519). In panels (A, B), “*G+*” or “*C+*” indicates carriers of the respective *G* or *C* allele in either the homozygous or heterozygous state, whereas “*TT*” or “*GG*” denotes homozygosity for the *T* or *G* allele, respectively. Group sizes and statistical tests were as follows: **(A)** *G +* vs. *TT*, *n* = 24 vs. *n* = 6, Mann–Whitney U test; **(B)** *C +* vs. *GG*, *n* = 15 vs. *n* = 15, Mann–Whitney U test; **(C)** *GGG* vs. *non-GGG*, *n* = 24 vs. *n* = 5, unpaired *t*-test; **(D)** *CTT* vs. *non-CTT*, *n* = 15 vs. *n* = 15, Mann–Whitney U test. Data are presented as box plots and expressed as **(A,B,D)** median ± interquartile range in panels and as **(C)** mean ± standard deviation in panel C; individual dots represent single observations.

Moreover, some relationships between the assessed *IL18* polymorphisms and biochemical parameters were seen. Although no statistically significant differences were detected, a trend toward lower CSF protein concentration (median: 54.00 vs. 80.70, *p* = 0.0730) was noted in carriers of the rs1946518 *G* allele, as compared to *TT* genotype (Figure 4A), which is consistent with the previously observed shorter distal CMAP latencies of peroneal motor fibers and may indicate less severe demyelinating changes. It was observed that the rs187238 *C* allele, as compared to *GG* genotype, was associated with higher protein concentration (median: 70.00 vs. 48.70, *p* = 0.0086, respectively; Figure 4B). Furthermore, The *GGG* haplotype was also associated with reduced CSF protein concentration (mean: 56.14 vs. 104.20, *p* = 0.0085; Figure 4C). In contrast, patients carrying the *CTT* haplotype exhibited significantly higher CSF protein levels (median: 70.00 vs. 48.70, *p* = 0.0086; Figure 4D).

**Figure 5:**
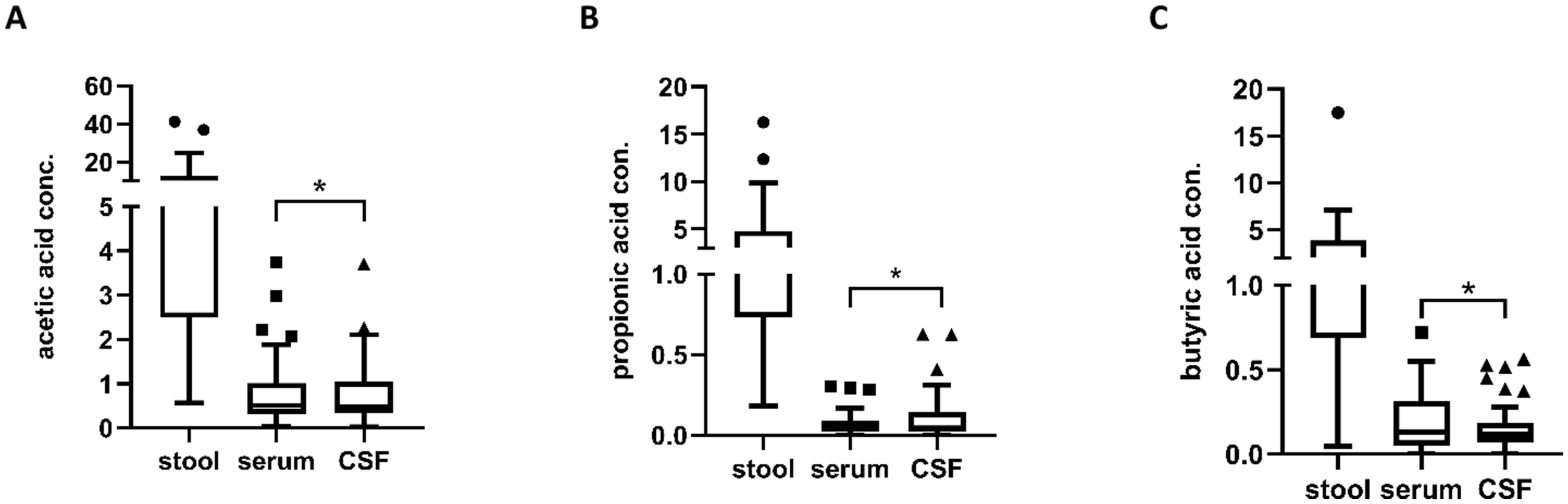
Differences in the concentrations of short-chain fatty acids: acetic **(A)**, propionic **(B)**, and butyric **(C)**; measured in stool (nmol/g), serum (nmol/μl), and cerebrospinal fluid (CSF; nmol/μl). Serum and CSF concentrations were equivalent for all three fatty acids, as demonstrated by two one-sided tests (TOST) for equivalence (*p* < 0.05 for equivalence), whereas stool levels were significantly higher. Group sizes for stool, serum, and CSF were *n* = 32, *n* = 44 and *n* = 48, respectively, for all three panels **(A–C)**, and group comparisons were performed using the Mann–Whitney *U* test. Data are presented as box plots and expressed as median ± interquartile range; individual dots represent single observations. Asterisks indicate statistically significant differences (*p* < 0.05); horizontal connectors on the plots denote the pairs of compartments with significant differences.

Concentrations of short-chain fatty acids (lactic, valeric, isovaleric, acetic, propionic, and butyric) were measured in stool, serum, and CSF. Lactic acid was detected in only two stool samples at low concentrations (0.149–0.366 nmol/g) and was undetectable in both serum and CSF. Isovaleric and valeric acids were readily detectable in stool but occurred only sporadically in serum and CSF. Accordingly, these three analytes were excluded from subsequent statistical analyses. A statistically significant difference was observed between stool and serum for all the fatty acids analyzed (*p* < 0.001), but not between serum and CSF. On the contrary, a TOST equivalence test confirmed that serum and CSF values are equivalent for all the fatty acids (*p* < 0.05) (Figures 5A-C).

**Figure 6:**
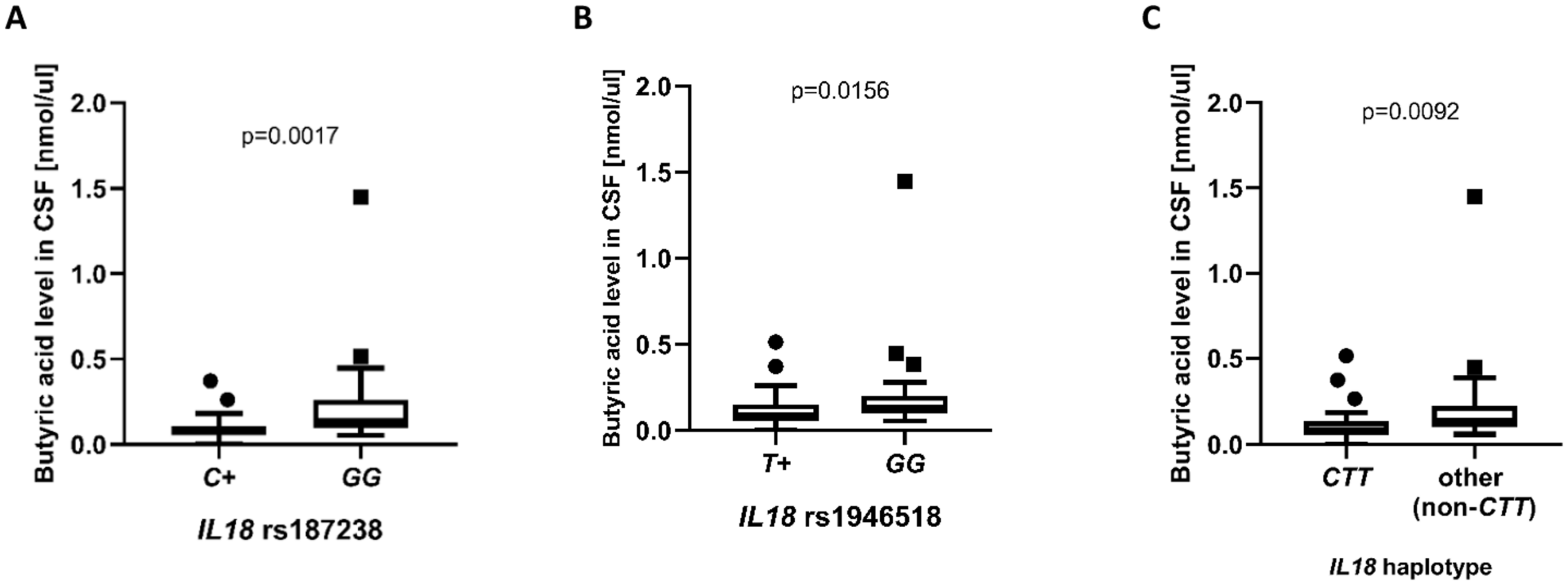
Cerebrospinal fluid butyrate concentrations (nmol/μl) in patients with different *IL18* polymorphic variants: rs187238 **(A)**, rs1946518 **(B)**, and the *CTT* haplotype (C; allele order: rs187238, rs1946518, rs1946519). In panels **(A)** and (B), “*T+*” and “*C+*” indicate carriers of the respective allele in either the homozygous or heterozygous state, whereas “*GG*” denotes homozygosity for the *G* allele. Group sizes were as follows: **(A)** *C +* vs. *GG*, *n* = 23 vs. *n* = 20, **(B)** *T +* vs. *GG*, *n* = 20 vs. *n* = 23, and **(C)** *CTT* vs. *non-CTT*, *n* = 21 vs. *n* = 22; all comparisons were performed using the Mann–Whitney U test. Data are presented as box plots and expressed as median ± interquartile range; individual dots represent single observations.

Additionally, we looked for any associations between SCFA concentrations and the *IL18* polymorphisms. No statistically significant associations were observed for any of the serum SCFA concentrations, and in CSF statistically significant associations were restricted to butyrate concentrations. Further analyses revealed that the rs187238 *C* (compared to *GG* genotype) and rs1946518 *T* (compared to *GG* genotype) alleles were associated with reduced CSF butyrate levels (median: 0.0780 vs. 0.1410 nmol/µl; *p* = 0.0017 and median: 0.0930 vs. 0.1340 nmol/µl, *p* = 0.0156, respectively) (Figures 6A,B). When looking at the haplotypes, patients carrying the *CTT* haplotype exhibited significantly lower CSF butyrate concentrations (median: 0.0800 vs. 0.1340 nmol/µl, *p* = 0.0092; Figure 6C).

## 5 Discussion

The diagnosis and treatment of CIDP remain challenging, as reflected in successive guideline revisions. Diagnosis still relies primarily on clinical features and electrophysiological testing, and the lack of definitive biological biomarkers continues to limit the sensitivity and specificity of current criteria (Van den Bergh et al., 2021; Brannagan, 2011; Eftimov et al., 2020). This is likely one of the reasons for the still-unsatisfactory response to currently used therapies (approximately 70%) (Mair et al., 2025). In this context, the identification of biomarkers that can aid differential diagnosis and guide treatment selection is a priority (Allen et al., 2021). Recent studies started to define the broader genetic architecture of CIDP, alongside earlier studies showing that certain variants may modulate treatment response (Iijima et al., 2009; Bozovic et al., 2023). One of the principal discovery tracks focuses on inflammatory cytokines, such as IL-6, IL-17, CXCL10, and TNF-*α*, with CIDP biology, and some studies report correlations with disability or disease activity (Mathey et al., 1999; Cutellè et al., 2024). IL-18 is a potent IFN-*γ* inducing cytokine involved in myeloid to lymphoid crosstalk and tissue barrier homeostasis. It has been detected in human inflammatory neuropathies: IL-18 expression is induced in acute inflammatory demyelinating polyneuropathy, and serum levels rise in immune neuromuscular conditions, highlighting the cytokine’s proximity to peripheral nerve inflammation. While CIDP differs from AIDP, these observations support IL-18’s broader participation in peripheral nerve immune biology (Jander and Stoll, 2001). In our cohort, however, IL-2, TNF-α and IL-18 concentrations did not show significant associations with disease course, and none of these analytes showed a pattern that would plausibly help to distinguish immune-mediated demyelinating neuropathy from diabetes-associated neuropathy, which is an important finding given the persistent diagnostic uncertainty in this area. Clinically and electrophysiologically, CIDP and demyelinating forms of diabetic sensorimotor polyneuropathy substantially overlap, and previous work has documented frequent diagnostic confusion and misclassification between these entities (Rajabally et al., 2017; Dunnigan et al., 2013b). Independent of CIDP, type 2 diabetes itself is now widely regarded as a chronic low-grade inflammatory disease, characterized by persistently increased inflammatory mediators in metabolic tissues and in the circulation, which may mask additional, superimposed cytokine changes attributable to CIDP (Donath and Shoelson, 2011; Herder et al., 2009; Herder et al., 2005). Against this pro-inflammatory background, our results suggest that conventional serum/CSF cytokines such as IL-2, TNF-α and IL-18 are unlikely to resolve the distinction between CIDP and diabetic neuropathy on their own, and that more specific imaging, electrophysiological and fluid biomarkers might be needed to aid in differentiation, as suggested, e.g., for nerve ultrasound and CSF sphingomyelin (Heiling et al., 2024; Capodivento et al., 2021). Electrophysiological testing remains central to CIDP diagnosis, complementing clinical criteria and anchoring case adjudication in current EAN/PNS guidance (Van den Bergh et al., 2021). Yet, despite decades of routine use, the strength and consistency of correlations between the severity of nerve conduction abnormalities and clinical impairment are variable across cohorts. Some studies demonstrate meaningful links. Bril et al. (2010) and Grüter et al. (2022) showed that changes in CMAP amplitudes correlate with the severity of symptoms on clinical scales; whereas purely demyelinating metrics show weaker or context-dependent associations. CSF analysis is a second pillar in the work-up. The 2021 EAN/PNS guideline treats CSF as supportive rather than decisive, emphasizing age-adjusted interpretation and cautioning against over-reliance on modest protein elevations. Population-based data likewise show that CSF protein rises with age and certain comorbidities, underscoring the need for calibrated thresholds (Van den Bergh et al., 2021; Fautsch et al., 2023). Although albuminocytologic dissociation is common in CIDP, reports on its relationship to disease severity are mixed. Cinar et al. (2013) demonstrated correlation between higher CSF protein and more severe weakness as well as broader conduction involvement, whereas Capodivento et al. (2021) find stronger clinimetric correlations for alternative fluid biomarkers (CSF sphingomyelin) than for total protein itself. Against this backdrop, our cohort adds a genetics informed perspective. We observed that carriage of *IL18* promoter–region variants bearing the *G* allele at rs187238/rs1946518/rs1946519 (haplotype *GGG*) was associated with shorter distal CMAP latency in the peroneal nerve and lower INCAT disability, with a parallel trend toward lower CSF protein (not reaching statistical significance). Notably, *GGG* carriers also showed a more pronounced reduction of ulnar SNAP amplitudes, suggesting a relative alignment of these alleles with sensory/axonal vulnerability over purely demyelinating delay. In contrast, *IL18* rs187238 Callele was associated with higher CSF protein without clear links to NCS severity or clinical scales. To date, the polymorphisms discussed above have been investigated across a range of autoimmune and infectious diseases, but to our knowledge, they have not been evaluated in CIDP cohorts (Chen et al., 2012; Su et al., 2017). We therefore acknowledge that the *IL18* promoter variants and haplotypes identified here are probably unlikely to be specific to CIDP and may instead represent more general modifiers of immune biology. If replicated in larger, longitudinal cohorts, their clinical utility may lie primarily in prognostic stratification, once a diagnosis of CIDP had been firmly established, rather than in screening for CIDP across the broader spectrum of autoimmune conditions. On the other hand, in everyday practice, the key diagnostic challenge is rarely to distinguish CIDP from other systemic autoimmune diseases but rather from diabetic polyneuropathy, an issue we have discussed in detail above, so that even non-specific genetic modifiers could still be clinically valuable if they help with differential diagnosis within the CIDP population. Taken together, these findings are consistent with the concept of genetic modifiers that tune inflammatory demyelination and barrier/biochemical readouts; however, they warrant replication in larger, stratified cohorts and cross-phenotype evaluation in other neuropathies to establish diagnostic specificity and prognostic utility.

As part of our integrated analysis, we included SCFAs as one class of bacterial metabolites that are known to change under conditions of gut dysbiosis (Zeng et al., 2019). The gut microbiome has become an intensely investigated field across autoimmune and neurological disorders, with a rapidly growing body of work implicating intestinal dysbiosis and microbially derived metabolites in human physiology and disease. Recent reviews and experimental studies outline how dysbiosis and bacterial products, particularly SCFA, shape systemic and neuroimmune pathways (Zhao et al., 2023; Silva et al., 2020; Colombo et al., 2021). Olsson et al. (2021) reported that in newly diagnosed relapsing–remitting MS, serum acetate was reduced versus healthy controls and inversely associated with IFN-*γ*, whereas butyrate and valerate correlated positively with pro-inflammatory cytokines. Within CIDP specifically, early exploratory studies suggest that the gut milieu may be altered. In an exploratory pilot study of IVIg-treated CIDP patients, Svačina et al. (2023) found a distinct gut microbiome with higher diversity and an enrichment of Firmicutes, which are typically associated with SCFA production. Separately, Fu et al. (2023) reported gut dysbiosis with an enrichment of opportunistic pathogens in CIDP patients. Neither of the above studies quantified SCFAs in serum or CSF. A pathophysiological rationale for considering SCFAs in CIDP is two-fold. First, preclinical work demonstrates that SCFAs can influence central barriers: germ-free mice exhibit increased blood–CSF barrier permeability that is rescued by microbial recolonization or SCFA supplementation (Xie et al., 2023). Foundational experimental studies also show that gut microbiota and specific SCFAs modulate tight-junction integrity at blood–brain barrier level (Xie et al., 2023; Braniste et al., 2014; Hoyles et al., 2018). Second, SCFAs are detectable in primate CSF and have been linked, through defined biological pathways, neuroprotection and neuroregeneration in the peripheral nervous system, most notably for propionate acting via free fatty acid receptors and histone acetylation (Deng et al., 2019; Grüter et al., 2023). Although direct human data remain limited. To our knowledge, SCFA profiling in CIDP had not been systematically evaluated prior to our work. In the present study, we confirmed the presence of acetate, propionate, and butyrate in human CSF. However, no significant differences were observed between serum and CSF concentrations, in either the CIDP cohort or controls, while observing the expected gradient between stool and serum. If confirmed in independent cohorts, serum measurements could constitute a practical surrogate for nervous system SCFA exposure, potentially reducing the need for lumbar puncture in future studies. Moreover, we observed that the *IL18* promoter alleles rs187238 *C* and rs1946518 *T*, and the *CTT* haplotype were associated with lower CSF butyrate, the same *C* allele were linked to higher CSF protein, although this did not translate into worse clinical scales or clearly demyelinating electrophysiology in our cohort and may represent a chance finding that warrants replication.

The principal limitation of our study is the small sample size, which reflects the rarity of CIDP and the single-center design of the study. A second important challenge was obtaining cerebrospinal fluid from the control group, i.e., from individuals presumed to be healthy. This substantially constrained the strength of our inferences, particularly genetic testing. Accordingly, the observed associations should be assessed in larger cohorts, ideally within multicentre studies. Another limitation is the absence of broader gut-microbiome profiling; in this work we examined only one class of bacterial metabolites (SCFAs). To achieve a more complete picture of the relationships among clinical course, blood/CSF biochemical markers, and the gut microbiome, future studies should include species-level characterization of intestinal bacteria.

## 6 Conclusion

In our cohort, variation within the *IL18* promoter, most notably the *GGG* haplotype (rs187238 *G*/rs1946518 *G*/rs1946519 *G*) was aligned with a phenotype indicative of a milder demyelinating burden (shorter distal CMAP latency, tendency to lower CSF protein) and lower disability. By contrast, the *CTT* haplotype was associated with higher CSF protein and lower CSF butyrate, but no correlations with clinical disability or NCS parameters were observed. We also demonstrated the presence of acetate, propionate, and butyrate in human CSF and observed comparable levels in serum and CSF, a pattern that, if validated, suggests serum assays could serve as a practical surrogate of nervous system SCFA exposure. Collectively, *IL18* haplotypes and SCFA indices appear to be biologically anchored stratifiers, deserving validation in multicentre, longitudinal cohorts that integrate electrophysiology with CSF/serum biomarkers and microbiome profiling.

## Data Availability

The data are stored in a password-protected repository and will be available at: https://cloud.hirszfeld.pl/index.php/s/i96AkqzA9b2kgnw; access will be granted by the corresponding author upon reasonable request.
